# Gut Microbiota-Derived Short Chain Fatty Acids Induce Circadian Clock Entrainment in Mouse Peripheral Tissue

**DOI:** 10.1038/s41598-018-19836-7

**Published:** 2018-01-23

**Authors:** Yu Tahara, Mayu Yamazaki, Haruna Sukigara, Hiroaki Motohashi, Hiroyuki Sasaki, Hiroki Miyakawa, Atsushi Haraguchi, Yuko Ikeda, Shinji Fukuda, Shigenobu Shibata

**Affiliations:** 1 Laboratory of Physiology and Pharmacology, School of Advanced Science and Engineering, Waseda University, Tokyo, Japan; 2 Waseda Institute for Advanced Study, Waseda University, Tokyo, Japan; 3 Institute for Advance Biosciences, Keio University, 246-2 Mizukami, Kakuganji, Tsuruoka 997-0052 Japan; 4 PRESTO, Japan Science and Technology Agency, 4-1-8 Honcho, Kawaguchi, Saitama 332-0012 Japan

## Abstract

Microbiota-derived short-chain fatty acids (SCFAs) and organic acids produced by the fermentation of non-digestible fibre can communicate from the microbiome to host tissues and modulate homeostasis in mammals. The microbiome has circadian rhythmicity and helps the host circadian clock function. We investigated the effect of SCFA or fibre-containing diets on circadian clock phase adjustment in mouse peripheral tissues (liver, kidney, and submandibular gland). Initially, caecal SCFA concentrations, particularly acetate and butyrate, induced significant day-night differences at high concentrations during the active period, which were correlated with lower caecal pH. By monitoring luciferase activity correlated with the clock gene *Period2 in vivo*, we found that oral administration of mixed SCFA (acetate, butyrate, and propionate) and an organic acid (lactate), or single administration of each SCFA or lactate for three days, caused phase changes in the peripheral clocks with stimulation timing dependency. However, this effect was not detected in cultured fibroblasts or cultured liver slices with SCFA applied to the culture medium, suggesting SCFA-induced indirect modulation of circadian clocks *in vivo*. Finally, cellobiose-containing diets facilitated SCFA production and refeeding-induced peripheral clock entrainment. SCFA oral gavage and prebiotic supplementation can facilitate peripheral clock adjustment, suggesting prebiotics as novel therapeutic candidates for misalignment.

## Introduction

The circadian clock is an internal timekeeping system, which anticipates daily changes and helps maintain homeostasis in mammals^1–3^. The molecular clock consists of daily oscillations of clock gene expression (e.g. *Period1/2*, *Clock*, *Bmal1, Cry1*, and *Rev-erbα*) in cells both in the brain and in the peripheral tissues, where it is called a peripheral clock. Mutation or knockout of clock genes in mice results in a disrupted sleep-wake cycle and increased disease prevalence, including metabolic syndrome, heart disease, cancer, and mental disorders^1,2,4,5^. Conversely, these diseases can also disrupt circadian clock function, and cause subsequent negative effects^2^. For instance, diet-induced obesity dampens sleep-wake rhythmicity and decreases the amplitude of clock gene expression rhythms in the white adipose tissue^6^. The fundamental mechanism of the circadian clock is entrainment of the clock phase to external cues, such as light and food^2^. Disorganized entrainment, such as jet lag, is related to disease progression. Thus, timing and composition of food are significant factors in keeping our circadian clock functioning appropriately, and the related study is termed “chrono-nutrition”^7^. Recent evidence suggests that scheduled access to food prevented diet-induced obesity or slowed down tumour development in mice^8,9^. In this study, we focused on prebiotic dietary fibre (cellobiose) as a candidate nutrient, which modulates food-induced peripheral clock entrainment.

The microbiome, which inhabits our distal gut, is thought to be an important symbiont for maintaining homeostasis and is a novel therapeutic target for multiple diseases including obesity, intestinal immune dysfunction, kidney disease, and autism^10–12^. Recent progress in ‘omics’ technologies for analysing the microbiome has revealed an interesting insight into the circadian clock. There is day-night difference in composition, localization, or function of the intestinal microbiome, which is dependent on the host’s feeding cycle^13–17^. In addition, jet lag disturbs the microbiome, called “dysbiosis”, and this can cause obesity^17^. One of the signalling pathways between microbiota and host tissue is the metabolites produced by the microbiota. Short-chain fatty acids (SCFAs) of the straight-chain 2–4 carbon variety, such as acetate, butyrate, and propionate, and organic acid (lactate), are produced from dietary fibre by fermentation, absorbed into the blood stream, and distributed to the whole body^11,18–20^. Recent evidence has shown that dietary fibre intake or SCFA supplementation has beneficial effects on inflammatory bowel disease, colon cancer, obesity, diabetes, and cardiovascular disease through several signalling pathways, including GPCR (Gpr41, Gpr43, Gpr109a, and Olfr78)-mediated pathways, and direct inhibition of histone deacetylase^11,18–20^. More recently, Leone *et al*.^16^ reported that butyrate synchronized *Per2/Bmal1* expression rhythm in cultured mouse hepatocytes, and the *Per2/Bmal1* expression ratio in the mouse liver was altered by intraperitoneal injection of butyrate. However, the concentration of butyrate used was higher (5 mM of butyrate in culture) than actual concentrations observed *in vivo* (~100 μM in peripheral tissue)^19,20^, and it is still unclear whether SCFA has an effect on the peripheral clock entrainment after oral injection of SCFA or dietary fibre intake. Here, we first investigated whether caecal SCFA and lactate concentrations are rhythmic. Next, we investigated the effect of orally gavaged SCFA and lactate on PER2::LUC rhythms^21^ in peripheral tissues such as the liver, kidney, and submandibular gland of antibiotic-treated, microbiota-reduced mice, with different doses or timings of injection. Next, we applied SCFA to mouse embryonic fibroblast (MEF) or cultured liver slices while monitoring PER2::LUC rhythms to determine a direct or indirect effect of SCFA to peripheral tissues. Then, in order to elucidate the effect of SCFA production after intake of dietary fibre on the peripheral circadian clock, we investigated whether refeeding of dietary fibre-containing diets produced an increase in the entrainment speed of peripheral clocks to food intake patterns.

## Results

### Daily rhythms of caecal SCFA and pH

**Figure 1:**
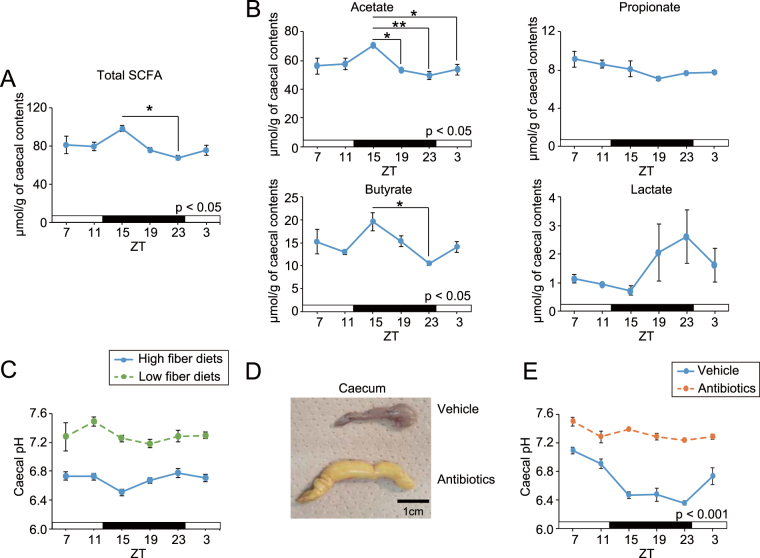
Daily fluctuations of caecal short chain fatty acid (SCFA), lactate content, and caecal pH in different conditions. (**A,B**) Caecal total SCFA content (**A**, acetate, butyrate, and propionate), each SCFA and lactate content (**B**, n = 3 tubes for SCFA measurement, 3 caecal samples were pooled in 1 tube). (**C**) Caecal pH was measured every 4 hours over 24 hours in ICR mice in *ad libitum* with high or low fibre diets (n = 10 for high fibre diets, n = 4 for low fibre diets in each time point). (**D,E**) Caecal picture (**D**) and pH (**E**) from vehicle or antibiotic-treated (at least a month by drinking) C57BL mice (n = 4 for each). All values are expressed as mean ± SEM. The P value of the one- or two-way ANOVA is indicated in the lower right side of each graph if significant. *p < 0.05, **p < 0.01, ***p < 0.001 (Tukey’s multiple comparisons test).

Because SCFA and lactate are metabolites of dietary fibre produced by microbiota, we expected their synthesis to have day-night differences and to depend on host activity and feeding behaviour. Therefore, we analysed caecal SCFA concentrations and pH over the course of a day. Significant rhythmicity was detected in total SCFA, acetate, butyrate, and pH in the caecum of intact ICR mice (Fig. 1A–C). Acetate and butyrate levels were higher at the beginning of the dark period, and consistently, caecal pH was lower at the same point because of the higher acidity. Lactate tended to have rhythmicity, but its concentration was 10 times lower than acetate or butyrate. Additionally, antibiotic treatment for a month or intake of a low fibre diet up-regulated baseline caecal pH and disrupted rhythmicity (Fig. 1C–E), suggesting that these treatments can reduce SCFA synthesis and that caecal pH measurement is a precise marker for SCFA production.

### Oral SCFA injection acutely changed the peripheral PER2::LUC phase

**Figure 2:**
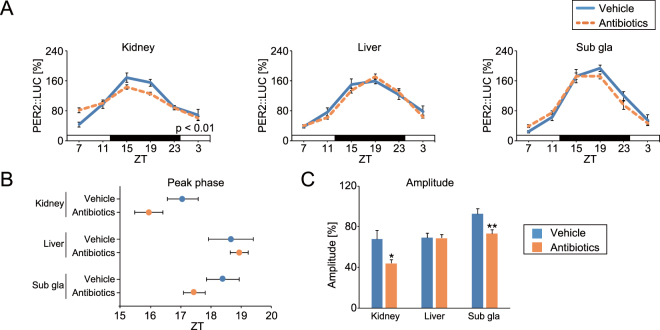
Effect of antibiotic treatment on peripheral PER2::LUC rhythms. Vehicle (water) or mixed antibiotics was administered to mice in drinking water over a month. (**A**) Average waveform of *in vivo* peripheral PER2::LUC imaging in the kidney, liver, and submandibular gland (sub gla). (**B,C**) Peak phase and amplitude of PER2::LUC rhythms. All the values are expressed as mean ± SEM. The number of mice used in this study is indicated in Table S1. More details on the statistics used is presented in Table S2. *p < 0.05, **p < 0.01, vs. vehicle gavaged group (t-test). P value of the two-way ANOVA is indicated in the lower right side of each graph if significant.

**Figure 3:**
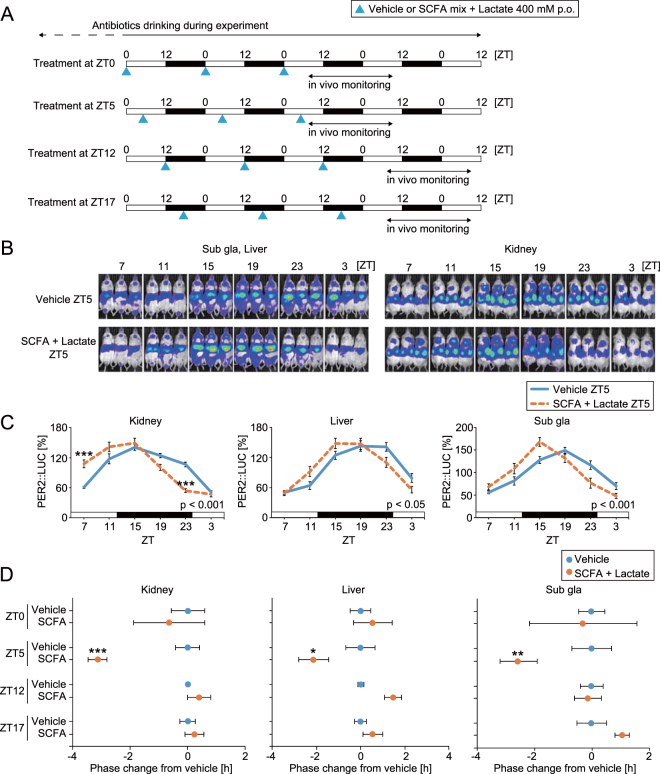
Administration of short chain fatty acids (SCFA) and organic acid (lactate) changed the phase of peripheral PER2::LUC rhythms with time-of-day dependency of treatment timing in antibiotic-induced microbiota depleted mice. (**A**) Experimental schedule. After drinking antibiotic-containing water for at least a month, mice were orally gavaged with vehicle (water) or SCFA + lactate mix (400 mM in each) (0.1 ml/10 g mouse weight) for 3 consecutive days at ZT0, 5, 12, or 17. Then, peripheral PER2::LUC bioluminescence rhythms were monitored every 4 hours for 24 hours starting at ZT7 after final injection. (**B,C**) Representative image or averaged waveform of *in vivo* peripheral PER2::LUC imaging in mice orally gavaged with vehicle or SCFA + lactate at ZT5. (**D**) Peak phase changes of PER2::LUC rhythm compared with the peak in the vehicle treatment group. The value of peak phase of the vehicle treatment was set as 0. All the values are expressed as mean ± SEM. The number of mice used in this study is indicated in Table S1. More details on the statistics is presented in Table S2. *p < 0.05, **p < 0.01, ***p < 0.001, vs. vehicle gavaged group (Tukey’s multiple comparisons or Mann Whitney test). P value of the two-way ANOVA is indicated in the lower right side of each graph if significant.

To exclude the effect of internal microbiota and their own SCFA production, mice were treated with antibiotics through drinking water for at least a month prior to the experiment, as in Fig. 1D and E. Of note, significant rhythmicity was detected in vehicle group (p < 0.005) but not in antibiotics group (p > 0.05) by cosinor fitting analysis. In addition, SCFA and lactate were not detected by HPLC analysis in the caecum of antibiotics-treated mice (n = 8, at ZT15). After antibiotic treatment, we measured peripheral PER2::LUC bioluminescence rhythms using *in vivo* imaging^21^. We found decreased amplitude in the kidney and submandibular gland, but not in the liver of antibiotic-treated mice (similar to previous findings^13^ in the liver; Fig. 2A and C). The peak phase of each tissue was not different from that of vehicle-treated mice (Fig. 2B). In the next experiment, we used our previously developed method in which several stimulations (e.g. re-feeding, nutrient injection, physical stress, or exercise), at specific times of the day for three consecutive days, caused acute phase shifting of peripheral PER2::LUC rhythms^22–24^. Mice were orally gavaged with vehicle (water) or SCFA and lactate mix (200 or 400 mM in each) at specific times (4 time points in a day, approximately 6-hour intervals) for three consecutive days. The peripheral PER2::LUC bioluminescence rhythms were then measured (Fig. 3A). The concentration of SCAF mix was chosen to be in line with that used in previous studies, and this is 2–3 times higher than the actual concentration in the mouse colon^25–27^. Since phase changes of the circadian clock are usually dependent on the stimulation time, we set 4 different experiments with treatment time at ZT0, 5, 12, or 17. Peak phases of PER2::LUC rhythms in the kidney, liver, and submandibular gland were significantly advanced by SCFA and lactate treatment at ZT5 (Fig. 1B–D) Only kidney was responded by SCFA treatment in lower dose (200 mM, Figure S1A). In addition, the similar phase shift was seen in the mice without antibiotic treatment in the kidney and liver (Figure S1B). On the other hand, SCFA and lactate treatment at ZT0, 12, or 17 did not change the phase of peripheral clocks. Thus, oral SCFA and lactate administration acutely changed PER2::LUC phase in the peripheral tissues with time-of-day treatment dependency.

To determine if oral SCFA and lactate treatment had a beneficial role in the circadian clock system, light-induced jet lag mice were prepared (Figure S2A). Treatment with SCFA and lactate at the beginning of newly shifted dark phase (8-hours advance of light-dark) for 5 consecutive days accelerated the phase entrainment of the PER2::LUC rhythms significantly in the kidney and slightly in the submandibular gland, but not in the liver (Figure S2B). Thus, increased SCFA and organic acid levels at the beginning of the active phase could help to recover from light shift-induced misalignment.

**Figure 4:**
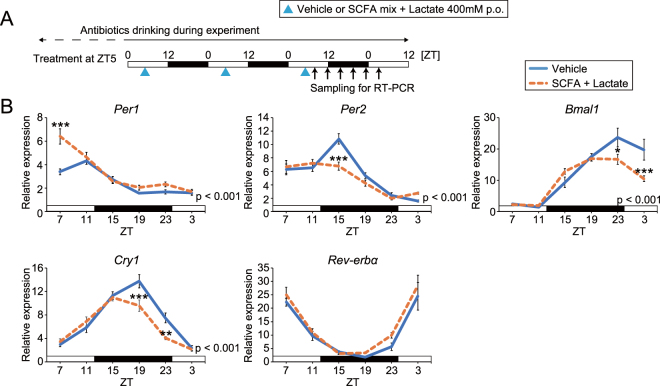
Administration of short chain fatty acids (SCFA) changed the phase of clock gene expression rhythms in the kidney of antibiotic-induced microbiota depleted mice. (**A**) Similar to Fig. 1A, antibiotic-induced microbiota depleted mice were orally gavaged SCFA + lactate mix (400 mM in each) at ZT5 for three consecutive days; then, kidneys were collected every 4 hours for 24 hours starting at ZT7 after the last injection. (**B**) Clock gene expression measured by RT-PCR. Peak phase analysis is shown in Table S3. Gene expression levels were normalized to *Gapdh*. All the values are expressed as mean ± SEM. P value of the two-way ANOVA is indicated in the lower right side of each graph if significant. **p < 0.01, ***p < 0.001, vs. vehicle gavaged group (Sidak’s multiple comparisons test).

To confirm whether SCFA and lactate-induced phase advance consistently occurred in the other clock genes, RT-PCR analysis was performed on kidney and submandibular gland tissue after vehicle or SCFA and lactate treatment at ZT5 (Figs 4 and S3). These tissues were selected, as these organs were more sensitive to SCFA and lactate, as evaluated by *in vivo* PER2::LUC. The phases of all clock genes (*Per1*, *Per2*, *Bmal1*, *Cry1*, and *Rev-erbα*) in the kidney and some (*Per2* and *Cry1*) in the submandibular gland were advanced by SCFA and lactate administration (Table S3), suggesting that not only *Per2* but also the molecular clock system was acutely entrained.

**Figure 5:**
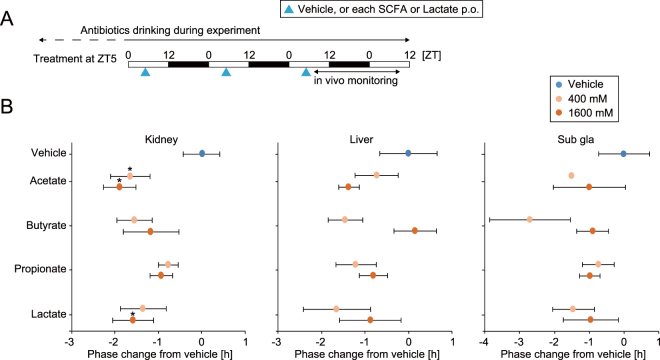
Administration of each short chain fatty acid (SCFA) changed the phase of peripheral PER2::LUC rhythms in antibiotic-induced microbiota depleted mice. (**A**) Similar to Fig. 1A, antibiotic-induced microbiota depleted mice were orally gavaged with each SCFA (400 or 1600 mM) at ZT5 for three consecutive days, and then we measured peripheral PER2::LUC every 4 hours for 24 hours starting at ZT7 after the last injection. (**B**) Peak phase difference compared to the peak of the vehicle only treatment is shown. The value of peak change of the vehicle-gavaged group was set as 0. All values are expressed as mean ± SEM. The number of mice used in this study is indicated in Table S1. *p < 0.05 vs. vehicle only treatment (t-test or Mann Whitney test).

To investigate the mechanism of the SCFA- and lactate-induced phase shift, the components of SCFA or lactate were singly gavaged into mice at ZT5 for 3 days (Fig. 5). To adjust the acid concentration as in the previous experiment, we used 400 or 1600 mM for each SCFA. Phase changes by each SCFA component or lactate administration were smaller than in mixed SCFA treatment, but we found that acetate and lactate have significant effects on phase advance in the kidney clock. Because there were no dose dependencies seen in the treatments, 400 mM of each treatment is sufficient to induce a change in the peripheral clocks. From these data, we could not find a single important component of SCFA which altered the peripheral clocks; instead, it seems that all components of SCFA and lactate contribute to the phase changes.

### SCFA induced phase changes in cultured MEFs or liver tissue

**Figure 6:**
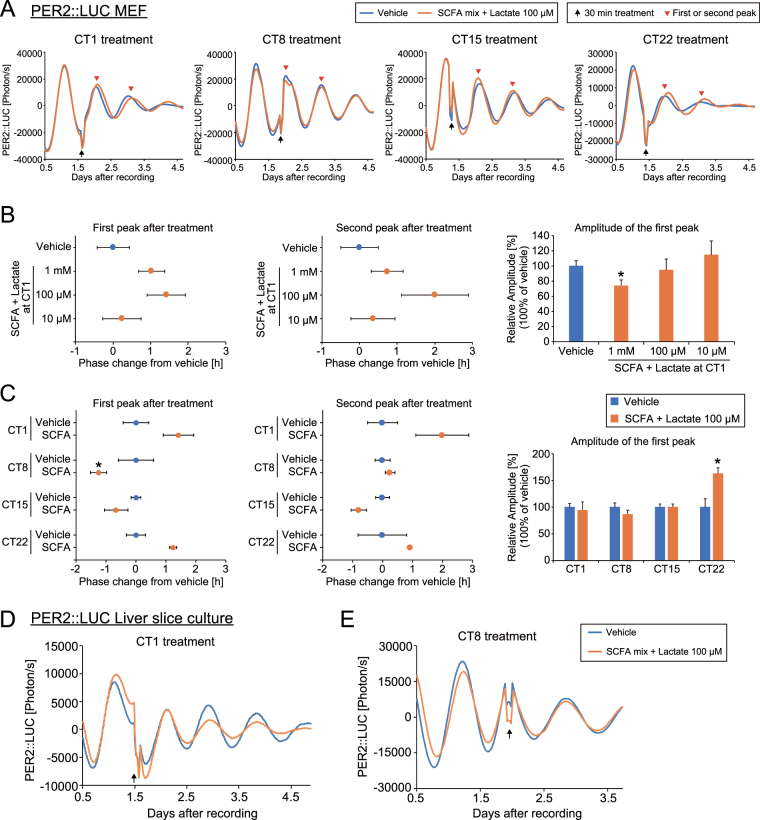
Application of short chain fatty acids (SCFA) failed to change PER2::LUC rhythms of mouse embryonic fibroblasts or cultured PER2::LUC liver slices, with dose and time-of-day dependency. (**A**) Representative PER2::LUC rhythms of each treatment timing. Circadian time (CT) 0 and CT12 were defined as the bottom and peak of the bioluminescence rhythm, respectively. Vehicle (water) or SCFA + lactate (100 μM) was applied to the media for 30 min (see methods section). Arrows indicate the treatment time and arrow heads indicate the first and second peaks we analysed. (**B,C**) Dose- or time-dependent phase changes of the first or second peak and amplitude of the first peak after SCFA treatment at CT1, 8, 15, or 22. Value of peak change of the vehicle treatment was set as 0. Value of amplitude of the vehicle treatment was set as 100. All the values are expressed as mean ± SEM (n = 4 in each group, except vehicle at CT22 n = 3). *p < 0.05 vs. vehicle only treatment (Mann Whitney test). (**D,E**) Representative PER2::LUC rhythms of each treatment timing in cultured liver slices. No difference was seen at the first peak after treatment between the vehicle and SCFA + lactate.

To understand the effect of SCFA and lactate *in vitro*, we used PER2::LUC MEF cells as a model of the peripheral clock^24,28^. PER2::LUC luminescence was recorded before and after transient application (30 min) at specific times. While it was not statistically significant, mixed SCFA and lactate treatment at Circadian time (CT, trough of PER2::LUC rhythm was set as CT0) 1 showed a tendency to cause phase delay in PER2::LUC rhythm, with dose dependency between 10 and 100 μM, but not at 1 mM SCFA and lactate, which instead caused decreased amplitude (Fig. 6A and B). Using 100 μM of SCFA, a trend of phase delay in the first and second peak were seen after treatment at CT1 and 22 (Fig. 6A and C). Significant phase advance was observed in the first, but not the second, phase after treating at CT8, meaning this effect was transient or weak. In addition, increased amplitude was seen in the treatment group after treatment at CT22. We also applied each SCFA or lactate to MEFs (Figure S4). The first peak of the acetate-treated group (100 μM at CT1) was delayed significantly, but the second peak was unchanged. In addition, there was no significant difference seen in MEFs treated at CT8. Therefore, only slight, and not statistically significant, effects of SCFA and lactate were seen on the phase shift of PER2::LUC in MEFs. To confirm this MEF result, cultured liver slices from PER2::LUC mice were treated with SCFA and lactate treatment at different treatment timings (CT1 and 8, Fig. 6D and E). Again, we did not observe any phase shift by this treatment. The *in vitro* study should show larger entrainment effects compared with *in vivo* study, because there are many other environmental entrainment factors (i.e. light-dark, activity, feeding, or body temperature) *in vivo* but not *in vitro*, and those factors could prevent the phase change by SCFA treatment. Thus, our *in vitro* data suggest that SCFA or lactate at physiological concentrations (i.e. 100 μM) do not affect molecular clocks directly.

### High fibre diets enhanced refeeding-induced peripheral clock entrainment

**Figure 7:**
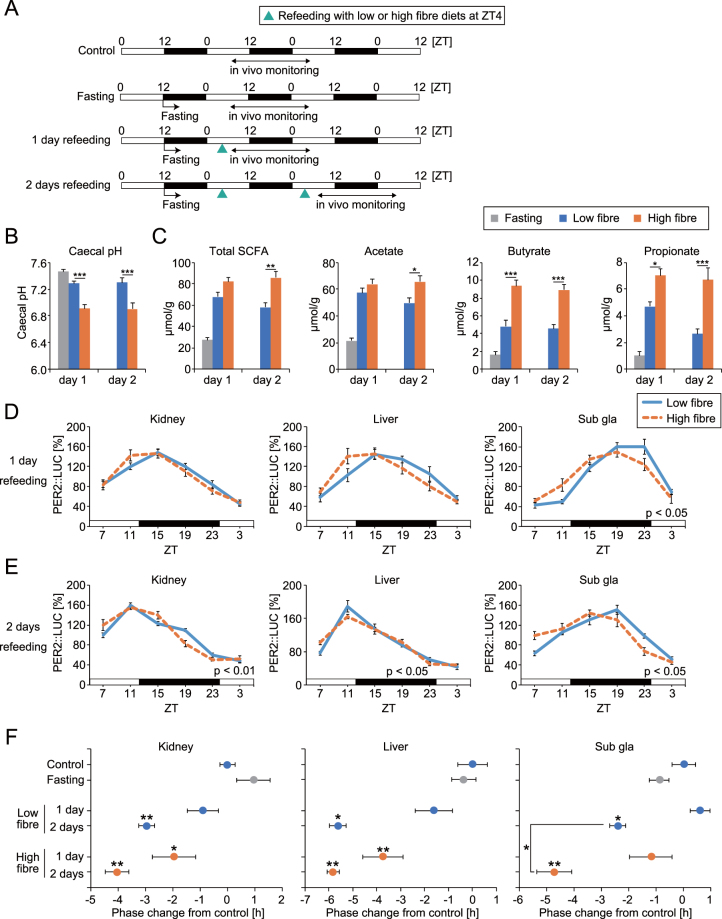
High fibre diets facilitated refeeding-induced phase resetting of peripheral PER2::LUC rhythms. (**A**) Experimental schedule. Control: Ad libitum feeding of low fibre diets. Fasting: Mice were fasted overnight. 1 day or 2 days refeeding: Mice were fasted from ZT12 and refed low or high fibre diets (1 g) at ZT4 for 1 day or 2 days. Peripheral PER2::LUC was monitored every 4 hours for 24 hours beginning at ZT7 after refeeding. (**B,C**) Caecal pH and SCFA content at 4 hours after refeeding of low or high fibre diets in overnight fasted mice (n = 6 for each, except n = 10 for pH of fasting group). (**D,E**) Averaged PER2::LUC rhythms in each condition. (**F**) Peak phase changes of PER2::LUC rhythm compared with the peak of the control group. The value of the peak phase of the control was set as 0. All values are expressed as mean ± SEM. The number of mice used in this study is indicated in Table S1. More detail on the statistics used is included in Table S2. *p < 0.05, **p < 0.01, ***p < 0.001, vs. vehicle or low fibre treatments (t-test or Tukey’s multiple comparisons test). P value of the two-way ANOVA is indicated in the lower right side of each graph if significant.

Refeeding after overnight fasting produces entrainment of peripheral clocks with phase advance^23,28^. From the result in Fig. 3, diet-induced production of SCFA may enhance this phase change. Here, using low (5% cellulose) and high (5% cellobiose) fibre diets we investigated the speed of refeeding induced peripheral clock entrainment. To quantify SCFA production from the microbiota, we used intact mice without antibiotic treatment in this study. Cellobiose but not cellulose can be digested by microbiota and increases bifidobacteria and SCFA production^29,30^. The SCFA concentration was higher at 4 hours after refeeding of high fibre diets compared with the low fibre diets or fasting group, with lower caecal pH (Fig. 7A–C). We could not detect the corresponding HPLC peak of lactate in each fasting and refeeding group. Significant phase changes of peripheral PER2::LUC rhythm were detected only in the high fibre diet group at 1 day after refeeding (Fig. 7D and F). In addition, larger phase changes were seen in the high fibre diet fed group’s peripheral clocks at 2 days after refeeding, compared with low fibre diets (Fig. 7E and F). These results indicate that the entrainment speed of peripheral clocks to feeding stimuli is enhanced by high fibre diet-induced SCFA production.

## Discussion

In the present study, we described four findings: (1) Caecal acetate and butyrate showed clear day-night fluctuations, with high amounts present during the active period. (2) Oral administration of mixed SCFA and lactate, or single administration of each SCFA or lactate in the middle of the day caused phase-advance of peripheral clocks *in vivo*. (3) Mixed SCFA and lactate administration to MEF cells or cultured liver tissue showed small but not statistically significant effects on clock entrainment. (4) Cellobiose containing diets accelerated refeeding-induced peripheral clock entrainment with increased caecal SCFA concentrations. Our findings suggest that microbiota-derived SCFA or lactate modulates the phase of host peripheral clocks *in vivo*.

Several known pathways could explain SCFA-induced peripheral clock entrainment. One is insulin-mediated clock entrainment through GPCRs. Insulin released from pancreatic β cells by feeding stimuli directly resets *Per2* gene expression rhythms in the liver and MEF cells^28,31^. In addition, we previously reported that DHA/EPA-containing diets enhanced food-induced entrainment through increased insulin by DHA/EPA-induced glucagon-like peptide 1 (GLP-1) secretion^32^. Recent studies also reported that SCFA binds to GPR41 and 43 expressed in intestinal L-cells, and induces peptide YY and GLP-1 secretion^33,34^. Altogether, SCFA-mediated insulin secretion could enhance peripheral clock entrainment. Other possible mechanism is sympathetic activation through GPR41. Propionate administration increases heart rate and energy expenditure through sympathetic activation via GPR41^19,35^. In addition, adrenergic or noradrenergic stimulation (i.e. adrenaline, noradrenaline, or adrenergic receptor agonist injection in mice) causes phase-shifting of peripheral clocks through activated transcription of a CRE site in the promotor of *Period* gene^23,36^. Another possibility is that SCFA-induced local pH change could cause phase resetting of the peripheral clocks. Kon *et al*.^37^ reported that alkali shock (pH + 0.4 of medium) reset circadian clocks *in vitro* through the TGF-β signalling pathway. Thus, pH-mediated clock resetting by SCFA may occur in the caecum or colon, and this possibility should be investigated in future studies. However, we did not detect significant pH changes in the SCFA-added medium in our MEF experiment (the concentration mimicked biological levels in the liver or kidney), suggesting that it is difficult to change local pH in peripheral tissues such as the liver. In contrast, another possibility is SCFA-induced epigenetic modulation. Butyrate and propionate are potent inhibitors of histone deacetylase (HDAC), and have the ability to control gene expression through this inhibition^11,19,20^. For instance, butyrate increases the differentiation of colonic regulatory T cells by changing histone H3 acetylation of the *Foxp3* promoter, and this results in protection against colitis development^38,39^. Additionally, circadian clock modulation by HDAC inhibitors (e.g. valproic acid or trichostatin A) in PER2::LUC MEF cells has been reported^40^. However, our *in vitro* study did not reveal any significant phase changes after SCFA administration in the same PER2::LUC MEF cells, suggesting direct HDAC inhibition by SCFA may not contribute to the phase changes seen *in vivo*. In addition, this low responsiveness of the *in vitro* data suggests that SCFA-induced phase shift of peripheral clocks might be mediated by second messengers, such as insulin or sympathetic nerves. Taken together, SCFA can modulate host peripheral clocks through several possible pathways, and these should be investigated in future studies.

Time-of-day dependent clock entrainment is one of the important features of the circadian clock system. Only SCFA injection in the middle of the day produced phase changes in peripheral clocks in the current study. Advancement of phase of the mouse peripheral clocks by stimulation in the middle of the day can be seen after arousal stimuli, including refeeding, exercise, sleep restriction, and caffeine injection^22,24,41^. These stimuli cause phase-delay if the stimulus occurs during the dark period, and this trend was also seen in this study in the liver or kidney after SCFA treatment at ZT12 or 17. The reason why SCFA can cause phase advancement in the daytime is that there is daily oscillation of SCFA production with higher amounts occurring during the dark period. Acute increase of SCFA concentration during the light period could be a more potent stimulation than during the dark period. Similar explanations can be seen in restraint stress-induced entrainment of peripheral clocks via activation of the sympathetic nervous system and glucocorticoid secretion because these pathways are more active during the active period compared to the resting period^3^. Large phase advances after restraint stress during the resting period was seen in mouse peripheral tissues^22^. Thus, it may be possible that mouse peripheral clocks show larger phase changes after SCFA injection during the daytime compared to injection during the night time.

In this study, we focused on the peripheral clocks instead of the central clock in the brain. This is because in our previous studies, acute (<3–5 days) entrainment stimuli such as refeeding, stress, or caffeine injection could not change the clock phase of the suprachiasmatic nuclei in the hypothalamus under normal light-dark conditions, which are the dominant stimuli in the suprachiasmatic nuclei^22,24,41^. However, we still do not know the effect of chronic or acute SCFA administration under constant darkness on central or peripheral clock entrainment. Recent evidence suggests that SCFA or microbiota affect brain function, such as in anxiety or depression^10^. In addition, probiotics have been reported to improve the circadian clock and sleep. Miyazaki *et al*.^42^ reported that mice fed heat-killed *Lactobacillus* increased their activity in the dark and increased non-rapid eye movement sleep during the light period. Thus, it is possible that microbiota-derived metabolites reach the brain, and future studies are needed to understand the effect of chronic SCFA administration on the circadian clock system in the brain.

We demonstrated clear day-night oscillation of caecal SCFA and pH in ICR mice with high SCFA amounts at beginning of the active period; similar caecal pH oscillation was consistently observed in C57BL6 mice. Daily oscillating SCFA rhythms in the caecum were reported previously, but here the peak phase of the different SCFAs were seen at different timings (butyrate, ZT2; propionate, ZT2; acetate, 2 peaks at ZT2 and ZT10)^16^. The reason why we detected different peak phases compared with this previous study is still unclear, but it might be because of the laboratory conditions, measurement protocol (GC-MS vs. HPLC), mouse conditions (conventionalized vs. SPF), diets, and mice feeding behaviour. Since immediate production of SCFA was seen in re-fed mice in our study, the rhythm of caecal SCFA might be caused by feeding rhythms. The rhythmicity of microbiome composition change is also regulated by feeding rhythm rather than host circadian clock genes. In fact, the daily rhythm of microbiome composition and serum metabolites were disrupted in clock gene knockout mice, but time-restricted feeding (i.e. daily food access in light phase) could restore those rhythms^13,17^. In addition, the enzyme of SCFA production (i.e. butyrate kinase, butyryl-CoA:acetate CoA-transferase) in the microbiota might have daily rhythms^16^. Thus, there is circadian rhythm in SCFA production in the caecum; however, further studies are needed to investigate the circadian regulation system of SCFA production.

Our current findings have shown for the first time that prebiotic fibre supplementation has a beneficial effect on clock adjustment. Our data present the hypothesis that prebiotics may be a possible means of relieving jet lag-induced misalignment and health disturbances, which needs to be assessed in future studies. Both the current and previous findings show that the microbiome plays an important role in maintaining host homeostasis, including the circadian clock system. Future studies are also needed to investigate whether there are similar effects of prebiotic nutrients on human circadian clocks.

## Methods

### Animals

Male ICR or C57BL/6J mice (Tokyo Laboratory Animals Science Co. Ltd., Tokyo, Japan) and male ICR background heterozygous PER2::LUC knock-in mice^43^ were used in this study. Animals were maintained and used according to the guidelines of the Committee for Animal Experimentation of the School of Science and Engineering at Waseda University and according to the laws of the Japanese government. The experiments were approved by the Committee for Animal Experimentation of the School of Science and Engineering at Waseda University (permission number; 2016-A044). Mice were maintained on a 12-h light/dark cycle (lights on at 08:00 h) at room temperature (23 °C ± 0.5 °C) and were provided with a standard MF diet containing high fibre (Oriental Yeast Co., Ltd., Tokyo, Japan, composition indicated in Table S5) and water *ad libitum* before the experiment. AIN-93M containing 5% cellulose was used as a low fibre diet, and AIN-93M containing 5% cellobiose (Tokyo Kasei Co. Tokyo, Japan) instead of cellulose was used as a high fibre diet^29,30^. The number of mice used in each experiment is shown in Table S1.

### Drugs

For antibiotic treatment, metronidazole (1 g/L), ampicillin sodium (1 g/L), neomycin sulphate (1 g/L), and vancomycin hydrochloride (0.5 g/L) were mixed into the drinking water. For SCFA treatment, sodium acetate trihydrate, sodium butyrate, sodium propionate, and sodium L-lactate solution (70%) were diluted in the water (vehicle). All chemicals were purchased from Wako Pure Chemical Industries, Ltd. (Osaka, Japan).

### *In vivo* recording of bioluminescence rhythms in peripheral tissues

Bioluminescence oscillations in peripheral tissues were monitored as previously described^21^. Briefly, mice were anaesthetized with a mixture of isoflurane (Mylan Inc., Tokyo, Japan) and concentrated oxygen. d-luciferin potassium salt (Promega, Madison, WI, USA) was injected subcutaneously (15 mg/kg) at the base of the neck between the shoulders. Dorsal and ventral side-up images were acquired with a 1-min exposure time at 8 min and 10 min after luciferin injection, respectively, using an *in vivo* imaging system (Perkin Elmer, Waltham, MA, USA). Images were obtained 6 times per day at 4-h intervals using the same mice. Mice were returned to their home cages between imaging sessions. Photon counts for each tissue were analysed using Living Image 3.2 software (Perkin Elmer). For each individual organ, the average daily photon/sec value was designated as 100% and used to represent daily bioluminescence rhythms. The peak phase, amplitude, and rhythmicity of normalized data were determined using the Single Cosinor Procedure program (Acro.exe version 3.5)^44^. Cut-off values for rhythmicity (goodness of fit value ≤ 0.1) were established to determine whether data were rhythmic or arrhythmic, and only rhythmic data were used for analyses of peak phase and average waveforms of normalized PER2::LUC rhythms. The number of samples that met these criteria is shown in Table S1.

### Real time RT-PCR

RNA was extracted from peripheral tissues using phenol (Omega Bio-Tek Inc., Norcross, GA, USA). Real-time RT-PCR was performed using the One-Step SYBR RT-PCR Kit (Takara Bio Inc., Shiga, Japan) with specific primer pairs (see Table S4) and a Piko Real PCR system (Thermo Fisher Scientific, Waltham, MA, USA). Primers were designed using Primer 3 software (produced by Steve Rozen and Helen Skaletsky). The relative expression levels of target genes were normalized to *Gapdh* expression. Data were analysed using the ΔΔCt method. A melt curve analysis was performed to identify non-specific products. The peak phase and rhythmicity were determined using the Single Cosinor Procedure program, and the results are shown in Table S3.

### *In vitro* recording of bioluminescence rhythms in mouse embryonic fibroblasts

The rhythmic expression of *Per2* was measured using a real-time LUC assay in mouse embryonic fibroblasts (MEFs) derived from PER2::LUC knock-in mice^24,28^. Bioluminescence was monitored once per minute over 10-min intervals with a dish-type luminometer (LumiCycle; Actimetrics, Wilmette, IL, USA). To synchronize circadian rhythm in fibroblasts, 200 nM dexamethasone (Sigma-Aldrich Co., MO, USA) was added to the cell culture medium and cells were incubated for 2 h at 37 °C. Then, the medium was replaced with Dulbecco’s modified Eagle’s medium (DMEM) containing 0.1 mM D-luciferin potassium salt (Promega, Madison, WI, USA) and 10% foetal bovine serum (FBS; Bio West, Kansas City, MO, USA). For transient treatment, MEFs were treated with control (water, 10 μl/1 ml medium/dish) or SCFA (10 μM to 1 mM) for 30 min at 37 °C at selected time points. The treatment timing was defined using Circadian time (CT; CT0 was set at the time of the trough of the PER2::LUC rhythm). After treatment, the reagent-containing culture medium was replaced with the previous medium, and bioluminescence was measured over 4 days, following which the first peak or second phase after treatment was analysed.

### Caecal pH and SCFA concentration measurement

Caecal pH was measured by inserting the glass tip of the electrode of a pH meter (Eutech Instruments, IL, USA) directly into the caecum. Caecal SCFA and organic acid concentrations were measured by ion-exclusion HPLC as previously described^45^. Pooled (n = 3) or single caecal concentrations were used for analysis in this study.

### Statistical analysis

Data were analysed using GraphPad Prism (version 6.03, GraphPad software, San Diego, CA, USA). Equal variance and normality tests were performed to select an appropriate statistical approach for each analysis. Parametric analyses were conducted using a 1-way or 2-way ANOVA with Tukey, Dunnett, or Student’s *t*-test post-hoc tests. Non-parametric analyses were conducted using a Kruskal-Wallis/Friedman test with Dunn or Mann-Whitney post-hoc tests. For ANOVA analyses, F and p values for each result are shown in Table S2. Data are expressed as the mean ± SEM. P < 0.05 was considered to indicate statistical significance.

## Electronic supplementary material

Supplemental information
