# Supplementary material for: Gut Microbiota-Derived Short Chain Fatty Acids Induce Circadian Clock Entrainment in Mouse Peripheral Tissue
Source: Sci Rep. 2018 Jan 23;8:1395. doi: 10.1038/s41598-018-19836-7 (PMC5780501; doi:10.1038/s41598-018-19836-7)
Supplement: Supplementary file 1 — Supplemental information [file 41598_2018_19836_MOESM1_ESM.pdf]

Supplemental information

Gut Microbiota-Derived Short Chain Fatty Acids Induce Circadian Clock Entrainment in Mouse Peripheral Tissue

Yu Tahara*[1,2], Mayu Yamazaki*[1], Haruna Sukigara[1], Hiroaki Motohashi[1], Hiroyuki Sasaki[1], Hiroki Miyakawa[1], Atsushi Haraguchi[1], Yuko Ikeda[1], Shinji Fukuda[3,4], and Shigenobu Shibata[1,†]

[1]Laboratory of Physiology and Pharmacology, School of Advanced Science and Engineering, Waseda University, Tokyo, Japan

[2]Waseda Institute for Advanced Study, Waseda University, Tokyo, Japan

[3]Institute for Advance Biosciences, Keio University, 246-2 Mizukami, Kakuganji, Tsuruoka 997-0052, Japan

[4]PRESTO, Japan Science and Technology Agency, 4-1-8 Honcho, Kawaguchi, Saitama 332-0012, Japan

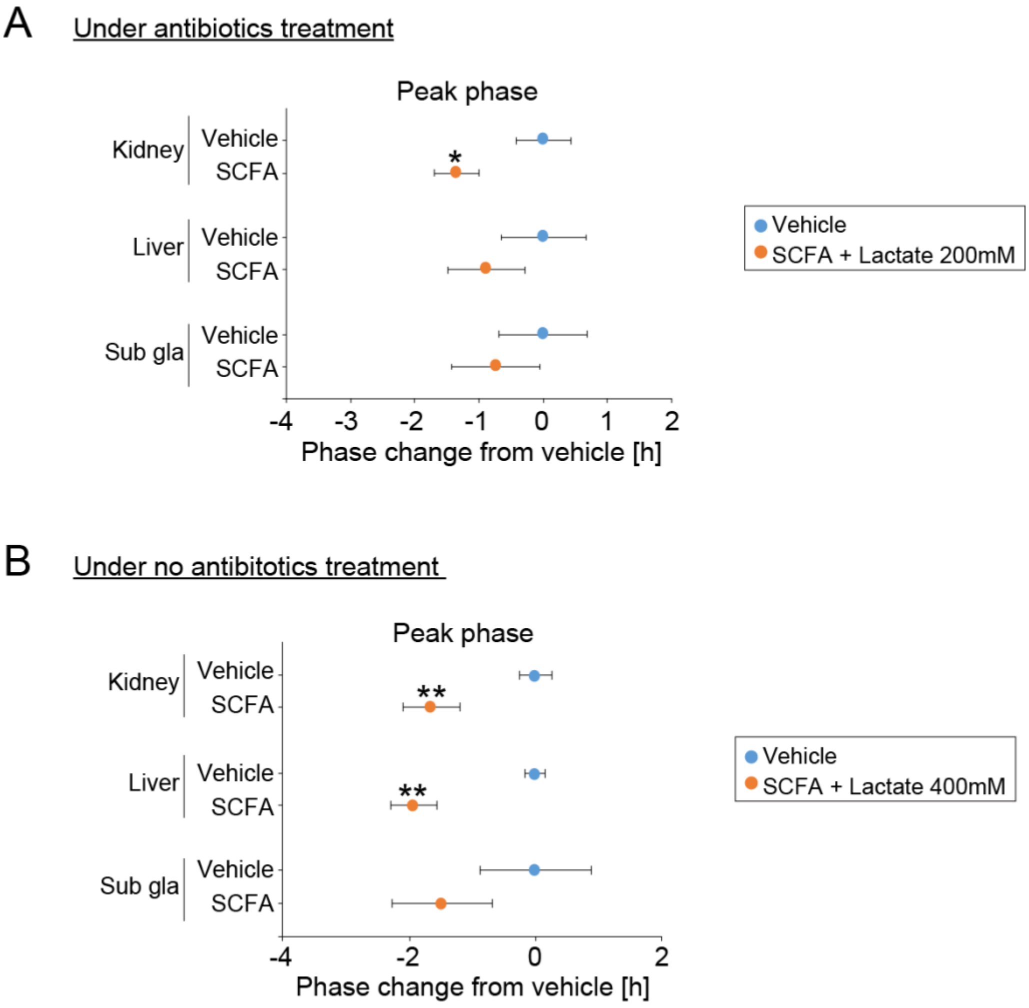

Figure S1. Administration of short chain fatty acids (SCFA) and organic acid (lactate) changed the phase of peripheral PER2::LUC rhythms (related to Figure 3). (A) Peak phase changes of PER2::LUC rhythms in the peripheral tissues. Mice were treated by vehicle or low dose of SCFA + lactate mix (200 mM) with a same schedule of Figure 3A. (B) Peak phase changes of PER2::LUC rhythms in the peripheral tissues. Mice were not treated with antibiotics. Mice were treated by vehicle or SCFA + lactate mix (400 mM) with a same schedule of Figure 3A. The value of peak change of the vehicle-injected group was set as 0. All the values are expressed as mean ± SEM. The number of mice used in this study is indicated in Table S1. More details on the statistics is presented in Table S2. *p < 0.05, **p < 0.01, vs. vehicle injected group (Mann Whitney test).

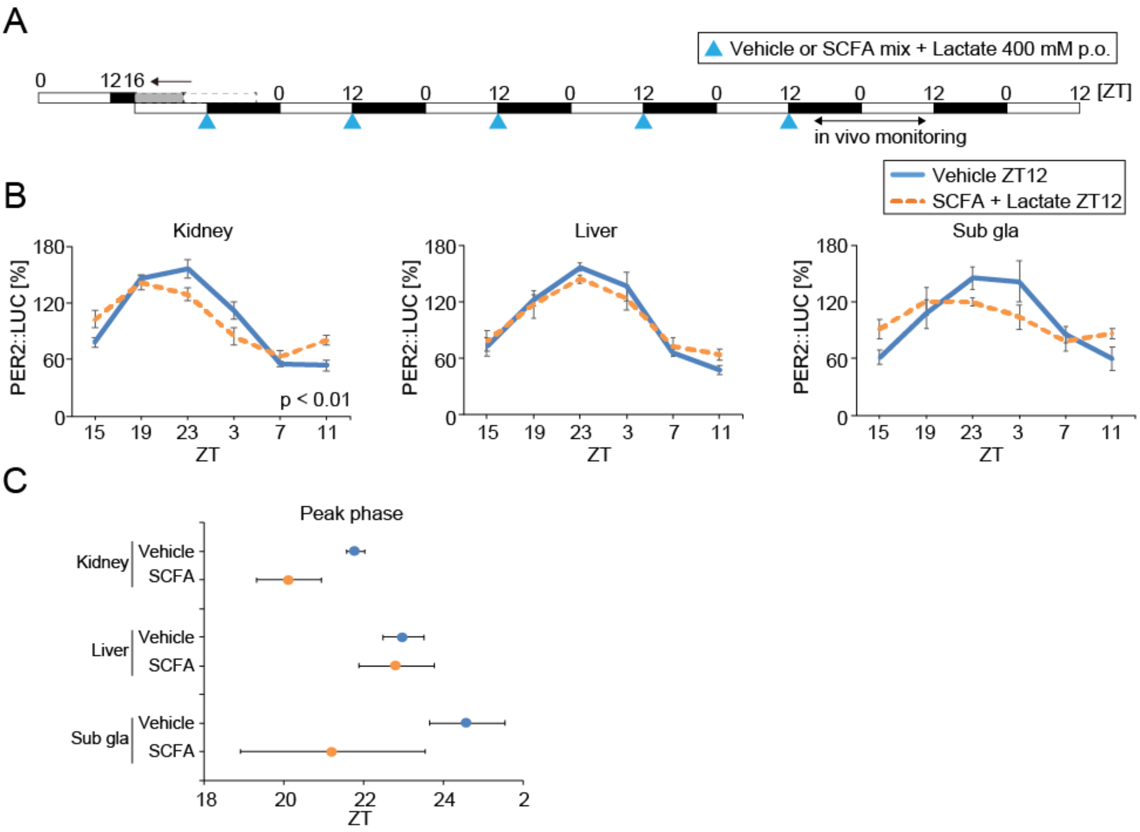

Figure S2. Administration of short chain fatty acids (SCFA) accelerated phase entrainment of peripheral PER2::LUC rhythms to 8-hour shifted new light-dark cycle. (A) Experimental schedule. 12 hour light-dark cycle was phase-advanced for 8 hours. Vehicle or SCFA + lactate was administered to mice at ZT12 for consecutive 5 days, then peripheral PER2::LUC rhythms were measured from ZT23. (B, C) Waveform (B) and peak phase (C) of PER2::LUC rhythms. All values are expressed as mean ± SEM. The number of mice used in this study is indicated in Table S1. More detail on the statistics is included in Table S2. P value of the two-way ANOVA is indicated in the lower right side of each graph if significant.

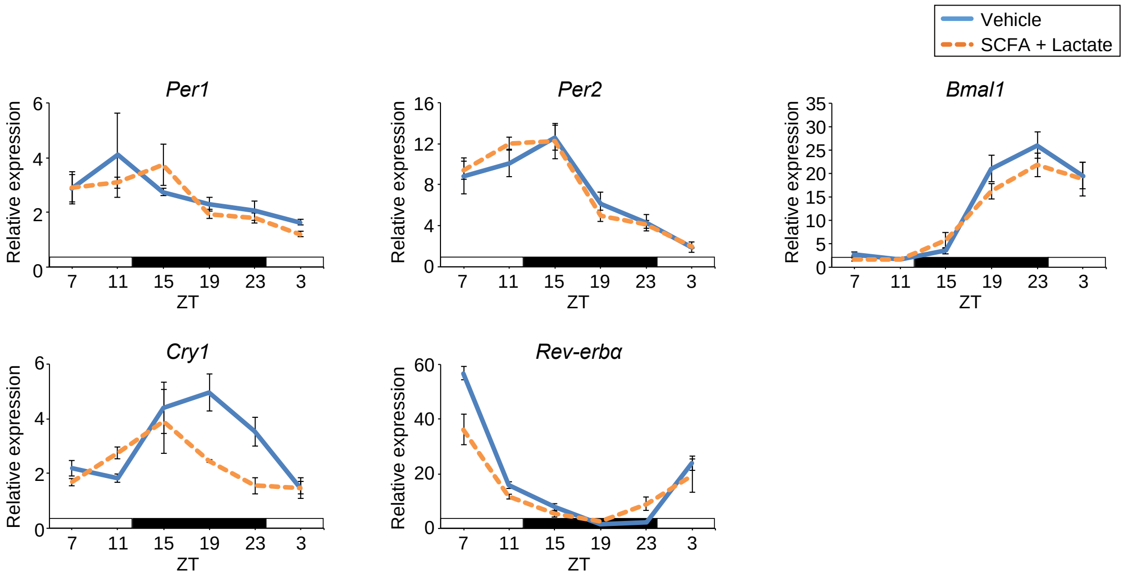

Figure S3. Administration of short chain fatty acids (SCFA) changed the phase of clock gene expression rhythms in the submandibular gland of antibiotic-induced microbiota depleted mice (related to Figure 4). (A) Similar to Fig. 1A, antibiotic-induced microbiota depleted mice were orally gavaged SCFA + lactate mix (400 mM in each) at ZT5 for three consecutive days; then, submandibular glands were collected every 4 hours for 24 hours starting at ZT7 after the last injection. (B) Clock gene expression measured by RT-PCR. Peak phase analysis is shown in Table S3. Gene expression levels were normalized to *Gapdh*. All the values are expressed as mean ± SEM.

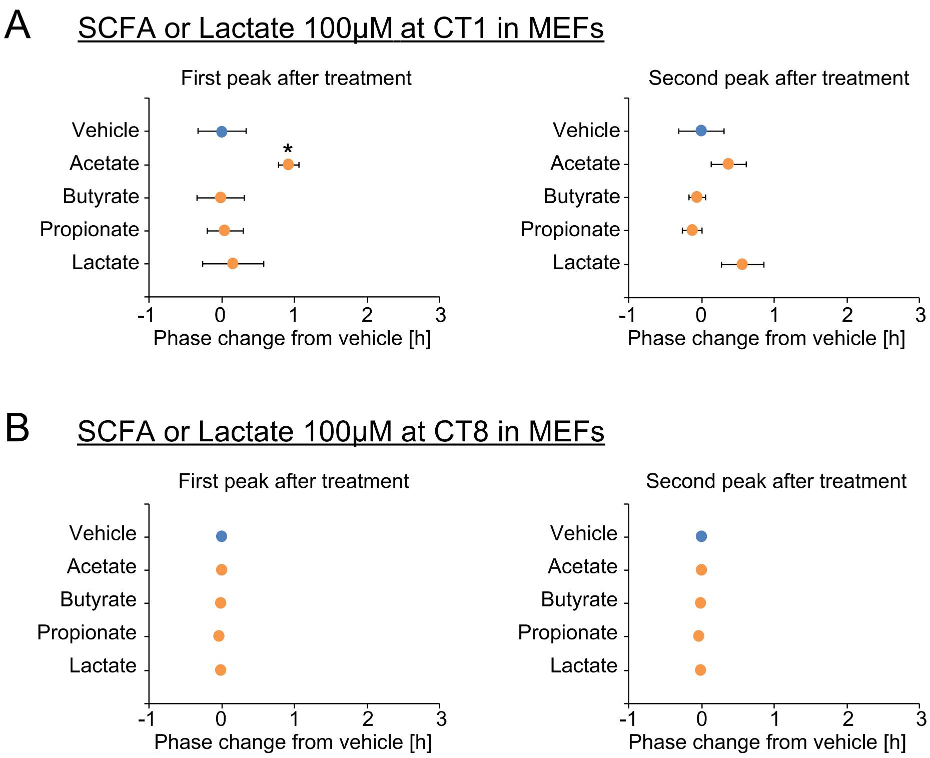

Figure S4. Administration of each short chain fatty acids (SCFA) or lactate in PER2::LUC mouse embryonic fibroblasts (related to Figure 6). Phase changes of the first or second peak after SCFA or lactate (100 μM) treatment at CT1 (A) or CT8 (B). Value of peak change of the vehicle treatment was set as 0. All the values are expressed as mean ± SEM (n = 4 in each group). *p < 0.05 vs. vehicle only treatment (t-test).

Table S1. Numbers of mice examined and tissues that met the rhythmicity criteria in each experimental group.

group name	number of mice	number of tissue samples showing rhythmic (rhythmic/total)			Figure No.
		kidney	liver	sub gla	
Vehicle	6	6/6	6/6	6/6	2
Antibiotics	8	8/8	8/8	8/8	2
Vehicle ZT0	3	3/3	3/3	3/3	3
SCFA ZT0	4	4/4	4/4	4/4	3
Vehicle ZT5	8	8/8	8/8	8/8	3, 5, S1
SCFA ZT5	9	7/9	9/9	9/9	3
Vehicle ZT12	3	3/3	3/3	3/3	3
SCFA ZT12	3	3/3	3/3	3/3	3
Vehicle ZT17	6	6/6	6/6	6/6	3
SCFA ZT17	5	5/5	5/5	5/5	3
Acetate 400 mM	5	5/5	5/5	2/5	5
Acetate 1600 mM	5	5/5	5/5	4/5	5
Butyrate 400 mM	4	4/4	3/4	4/4	5
Butyrate 1600 mM	5	5/5	5/5	5/5	5
Propionate 400 mM	5	5/5	5/5	5/5	5
Propionate 1600 mM	5	5/5	5/5	5/5	5
Lactate 400 mM	5	4/5	4/5	5/5	5
Lactate 1600 mM	7	6/7	7/7	7/7	5
Control	6	5/6	5/6	6/6	7
Fasting	5	5/5	5/5	5/5	7
Low fiber 1 day	6	6/6	6/6	6/6	7
Low fiber 2 days	6	6/6	3/6	5/6	7
High fiber 1 day	6	6/6	6/6	6/6	7
High fiber 2 days	6	6/6	6/6	6/6	7
SCFA 200 mM	8	8/8	5/8	6/8	S1
Vehicle (no antibiotics)	4	4/4	4/4	4/4	S1
SCFA ZT5 (no antibiotics)	5	5/5	5/5	5/5	S1
Vehicle with light shift	5	5/5	5/5	4/5	S2
SCFA with light shift	9	7/9	7/9	7/9	S2

Table S2. ANOVA results.

Figure No.	detail	ANOVA	main effect A			main effect B			Interaction effect	
			factor	F	P-value	factor	F	P-value	F	P-value
1A	Total SCFA	one-way	time	F (5, 12) = 7.78	p < 0.01					
1B	Acetate	one-way	time	F (5, 12) = 4.59	P < 0.05					
1B	Butyrate	one-way	time	F (5, 12) = 3.75	P < 0.05					
1B	Propionate	one-way	time	F (5, 12) = 1.84	P = 0.17					
1B	Lactate	one-way	time	F (5, 12) = 1.37	p = 0.30					
1D	pH in ICR	two-way	time	F (5, 72) = 2.45	p < 0.05	treatment	F (1, 72) = 218.3	p < 0.001	F (5, 72) = 1.219	p = 0.282
1E	pH in C57BL	two-way	time	F (5, 36) = 7.61	p < 0.001	treatment	F (1, 36) = 332.9	p < 0.001	F (5, 36) = 7.615	p < 0.001
2A	Kdiney	two-way repeated	time	F (5, 60) = 48.12	p<0.001	treatment	F (1, 12) = 0.506	p = 0.903	F (5, 60) = 4.719	p < 0.01
2A	Liver	two-way repeated	time	F (5, 60) = 57.2	p<0.001	treatment	F (1, 12) = 1.371	p = 0.264	F (5, 60) = 0.936	p = 0.464
2A	Sub gla	two-way repeated	time	F (5, 60) = 85.66	p<0.001	treatment	F (1, 12) = 1.371	p = 0.264	F (5, 60) = 1.44	p = 0.223
3C	Kdiney	two-way repeated	time	F (5, 65) = 50.93	p < 0.001	treatment	F (1, 13) = 1.090	p = 0.316	F (5, 65) = 12.46	p < 0.001
3C	Liver	two-way repeated	time	F (5, 75) = 35.55	p < 0.001	treatment	F (1, 15) = 0.045	p = 0.834	F (5, 75) = 3.14	p < 0.05
3C	Sub gla	two-way repeated	time	F (5, 75) = 30.87	p < 0.001	treatment	F (1, 15) = 1.775	p = 0.203	F (5, 75) = 5.31	p < 0.001
4B	Per1	two-way	time	F (5, 71) = 45.68	p < 0.001	treatment	F (1, 71) = 18.42	p < 0.001	F (5, 71) = 7.30	p < 0.001
4B	Per2	two-way	time	F (5, 72) = 45.53	p < 0.001	treatment	F (1, 72) = 3.05	P = 0.08	F (5, 72) = 5.71	p < 0.001
4B	Bmal1	two-way	time	F (5, 71) = 50.59	p < 0.001	treatment	F (1, 71) = 5.96	p < 0.05	F (5, 71) = 4.97	p < 0.001
4B	Cry1	two-way	time	F (5, 72) = 63.09	p < 0.001	treatment	F (1, 72) = 7.52	p < 0.01	F (5, 72) = 4.72	p < 0.001
4B	Rev-erba	two-way	time	F (5, 72) = 41.87	p < 0.001	treatment	F (1, 72) = 2.85	p = 0.09	F (5, 72) = 0.33	P = 0.88
7D	Kdiney	two-way repeated	time	F (5, 50) = 39.01	p < 0.001	treatment	F (1, 10) = 1.589	p = 0.236	F (5, 50) = 1.01	p = 0.421
7D	Liver	two-way repeated	time	F (5, 50) = 21.13	p < 0.001	treatment	F (1, 10) = 2.239	p = 0.166	F (5, 50) = 2.01	p = 0.093
7D	Sub gla	two-way repeated	time	F (5, 50) = 43.56	p < 0.001	treatment	F (1, 10) = 0.038	p = 0.850	F (5, 50) = 3.17	p < 0.05
7E	Kdiney	two-way repeated	time	F (5, 50) = 89.58	p < 0.001	treatment	F (1, 10) = 3.772	p = 0.081	F (5, 50) = 3.88	p < 0.01
7E	Liver	two-way repeated	time	F (5, 35) = 93.45	p < 0.001	treatment	F (1, 7) = 2.337	p = 0.170	F (5, 35) = 2.82	p < 0.05
7E	Sub gla	two-way repeated	time	F (5, 45) = 30.97	p < 0.001	treatment	F (1, 9) = 0.373	p = 0.556	F (5, 45) = 3.47	p < 0.05
S2	Kdiney	two-way repeated	time	F (5, 45) = 41.62	p < 0.001	treatment	F (1, 9) = 0.173	p = 0.686	F (5, 45) = 4.559	p < 0.01
S2	Liver	two-way repeated	time	F (5, 55) = 21.23	p < 0.001	treatment	F (1, 11) = -0.107	p > 0.999	F (5, 55) = 0.7618	p = 0.581
S2	Sub gla	two-way repeated	time	F (5, 45) = 4.11	p < 0.01	treatment	F (1, 9) = 2.93	p < 0.01	F (5, 45) = 1.181	p = 0.333

Table S3. Cosinor analysis of RT-PCR data.

Figure No.	Group	Vehicle		SCFA		
		Goodness of fit	Achrophase (hr)	Goodness of fit	Achrophase (hr)	Phase change (hr)
2	Per1	0.03	10.6	0.1	9	-1.6
2	Per2	0.04	13.4	0.01	11.8	-1.6
2	Bmal1	0.01	22.6	0.01	20.6	-2
2	Cry1	0.01	17.4	0.01	15.8	-1.6
2	Rev-erba	0.07	7	0.07	3	-4
S3	Per1	0.065	11.8	0.05	12.6	0.8
S3	Per2	0.035	13	0.027	12.2	-0.8
S3	Bmal1	0.016	23	0.019	23	0
S3	Cry1	0.038	18.2	0.03	14.6	−3.6
S3	Rev-erba	0.069	7	0.064	7	0

Table S4. Primer sequences for RT-PCR analyses.

Gapdh	5'-tggtgaaggtcggtgtgaac-3'
	5'-aatgaaggggtcgttgatgg-3'
Per1	5'-caagtggcaatgagtccaacg-3'
	5'-cgaagtttgagctcccgaagtg-3'
Per2	5'-tgtgtgcttacacgggtgtccta-3'
	5'-acgtttggtttgcgcatgaa-3'
Bmal1	5'-ccacctcagagccattgataca-3'
	5'-gagcaggtttagttccactttgtct-3'
Cry1	5'-atccgctgcgtctatatcctc-3'
	5'-cccgaatcacaaacagacg-3'
Rev-erbα	5'-cttccgtgacctttctcagc-3'
	5'-cagctcctcctcggtaagtg-3'

Table S5. Nutrient concentrations of MF diets

	MF (100g)
Moisture	7.9 g
Crude protein	23.1 g
Crude fat	5.1 g
Crude ash	5.8 g
Crude fibre	2.8 g
soluble nitrogen free extract	55.3 g
Calorie	359 kcal
